# The acute leukocyte and cytokine response of older adults to resistance exercise in normobaric hypoxia

**DOI:** 10.5114/biolsport.2023.116005

**Published:** 2022-06-01

**Authors:** Giselle Larissa Allsopp, Alex Bernard Addinsall, Garth Stephenson, Faiza Basheer, Paul Adrian Della Gatta, Samantha May Hoffmann, Aaron Paul Russell, Craig Robert Wright

**Affiliations:** 1 Institute for Physical Activity and Nutrition, School of Exercise and Nutrition Sciences, Deakin University, Victoria, Australia; 2 Department of Physiology and Pharmacology, Karolinska Insitutet, 171 77 Stockholm, Sweden; 3 School of Medicine, Deakin University, Geelong, Victoria, Australia; 4 Institute for Mental and Physical Health and Clinical Translation (IMPACT), Deakin University, Geelong, Victoria, Australia; 5 Centre for Sport Research (CSR), School of Exercise and Nutrition Sciences, Deakin University, Victoria, Australia

**Keywords:** Simulated altitude, White blood cells, Ageing, Strength training, Immune, Senescence, Lymphocytes

## Abstract

Ageing causes a decline in leukocyte function and blunted leukocyte responses to resistance exercise. Systemic hypoxia exposure augments the leukocyte response to resistance exercise in young adults, yet this response remains uncharacterised in older adults. This study characterised the effects of normobaric hypoxia on the acute leukocyte and inflammatory cytokine responses to resistance exercise in older adults. We recruited 20 adults aged 60–70 years to perform an acute bout of resistance exercise in normobaric hypoxia (FiO_2_ 14.4%; n = 10) or normoxia (FiO_2_ 20.93%; n = 10). Participants completed 4 × 10 repetitions of lower and upper body exercises at 70% of their predicted 1-repetition maximum. Venous blood was sampled before and up to 24 hours post-exercise to quantify neutrophils, lymphocytes, monocytes, eosinophils, basophils and cytokines (IL-1β, IL-4, IL-6, IL-8, IL-10, TNFα). Flow cytometry was used to classify lymphocytes as T (CD4^+^ helper and CD8^+^ cytotoxic), B and NK cells, in addition to the expression of the senescence marker CD45RA on T cells. The hypoxic group showed a larger lymphocyte response over the 24 hours post-exercise compared to the normoxic group (p = 0.035). Specifically, there were greater concentrations of CD4^+^ T helper cells following hypoxic exercise compared to normoxia (p = 0.046). There was also a greater proportion of CD45RA^+^ CD4^+^ T helper cells, suggesting that the cells were more senescent (p = 0.044). Hypoxia did not impact any other leukocyte population or cytokine following exercise. Normobaric hypoxia increases the lymphocyte response to an acute bout of resistance exercise in older adults.

## INTRODUCTION

The American College of Sports Medicine (ACSM) recommends that older adults perform regular bouts of resistance exercise to maintain their muscle health and functional independence [1]. Each acute bout of resistance exercise causes a rapid increase in leukocyte (white blood cell) populations and inflammatory cytokines in the systemic circulation for up to 24 hours post-exercise [2]. These leukocyte and cytokine populations likely play a role in resistance to pathogens in the hours post-exercise, with some leukocytes migrating to peripheral tissues such as skeletal muscle to aid in repair [3]. Older adults likely have a blunted systemic leukocyte response to resistance exercise [4] that may impact innate and adaptive immunity.

Resistance exercise impacts the five leukocyte subpopulations in the hours post-exercise; neutrophils, lymphocytes, monocytes, eosinophils and basophils [2]. Circulating neutrophils typically peak between 90 and 120 minutes after resistance exercise [5, 6], with monocytes typically elevated beyond 120 minutes [7]. Although there are few studies on the leukocyte responses of older adults to resistance exercise, it appears that their neutrophil response to damaging exercise (downhill running) is blunted compared to young adults up to 8 hours post-exercise [8]. Hulmi et al (2010) did not show a blunted neutrophil response in older adults compared to younger adults following a heavy resistance exercise bout, however blood was sampled up to 30 minutes and 48 hours post-exercise, and therefore the peak in neutrophil concentration was likely not captured [9]. The exercise-induced neutrophilia likely plays several important roles, including the control oxidative stress [10], pathogen removal by degranulation [11] and interaction with other leukocytes such as monocytes [12]. The increase in monocytes following an acute bout of resistance exercise is shorter in duration in older adults [4], possibly reducing their capacity to contribute to immune defence.

In healthy young adults, lymphocytes are typically elevated approximately 2-fold during and immediately post-resistance exercise (lymphocytosis), followed by a drop below resting levels by two hours post-exercise (lymphocytopenia) [2]. The lymphocytopenia in the hours following resistance exercise could be caused by the sequestering of lymphocytes back into tissues such as the lungs and lymphoid tissues, in addition to damaged muscle tissue [13]. The literature is divided on whether the lymphocytopenia signifies reduced resistance to infection (the ‘open window’) or rather enhanced immunosurveillance in the peripheral tissues where the lymphocytes redeploy [14, 15]. In older adults, the peak in lymphocyte concentration immediately following intense resistance exercise is potentially blunted compared to young adults [9, 16]. A coordinated lymphocyte response is vital to effectively respond to pathogens [17, 18], therefore this blunted lymphocyte response may signify an increased risk of infection and illness in older adults. It is currently unclear whether ageing causes a more pronounced lymphocytopenia in the hours post-exercise due to the scarcity of research and the lack of frequent blood sampling 2–3 hours post-exercise when lymphocytopenia is detectable.

The inflammatory cytokine response to resistance exercise is also dysregulated in older adults. For example, the systemic response of interleukin-6 (IL-6) following a bout of damaging eccentric exercise was less pronounced in older adults compared to younger adults [19]. The coordinated and proportionate signalling of inflammatory cytokines is vital for the effective coordination of the immune response [20].

The reasons for the dysregulation immune and cytokine responses to resistance exercise with ageing are not fully clear. In healthy young individuals, the acute increase in circulating leukocytes is likely caused by the exercise-induced increase in catecholamines and glucocorticoids that bind to receptors present on leukocytes [21–23]. Other factors contributing to the normal recruitment of leukocytes to the systemic circulation includes an increase in haemodynamic shear stress that reduces the adhesion of leukocytes to the endothelial lining of blood vessels [24]. With advancing age, contributors to leukocyte ageing (termed immunosenescence) may include the persistent exposure to pathogens across the lifespan and thymic involution that reduce the number of naїve T lymphocytes and their ability to respond to novel pathogens (see review; [25]). Ageing is also associated with altered concentrations of hormones such as cortisol, adrenaline and growth hormone [26, 27] that normally stimulate leukocyte populations during exercise [22, 28]. Given that the blunted immune response to resistance exercise in older adults may reduce their resistance to pathogens, interest is growing in strategies that reverse this blunted response.

Systemic hypoxia is a potent mobiliser of leukocyte populations, yet the combined effects of resistance exercise and systemic hypoxia have not been investigated in the context of ageing. In healthy young individuals, acute exposure to hypoxia for twenty minutes increases the number and function of circulating NK cells and increases monocyte concentrations [29]. These responses may confer increased resistance to infection. In combination with resistance exercise, systemic hypoxia is typically used with the aim of improving the training adaptations in young adults [30, 31]. In older adults, three studies have investigated if hypoxia can improve muscular strength, muscle mass and aerobic capacity responses to resistance training [32, 33, 34]. One of those studies reported greater muscle mass and muscle function responses in hypoxia in obese older adults [34] whilst two studies showed equivalent training adaptations in healthy older adults between hypoxia and normoxia [32, 33]. We recently characterised the leukocyte response following moderate-intensity resistance exercise in systemic hypoxia (fraction of inspired oxygen (FiO_2_) 14.4%) in young adults [35]. Participants in the hypoxic group showed a greater concentration of circulating neutrophils 120 and 180 minutes following the acute bout of resistance exercise, compared to normoxic controls. Although the mechanisms mediating this response are unknown, this result highlights the potential application of hypoxic training in older adults to reverse the blunted leukocyte response to exercise. Any improvements in the leukocyte response of older adults to exercise may confer increased resistance to pathogens in the hours post-exercise. Therefore, this study aimed to characterise and compare the acute leukocyte responses of older adults to a single bout of resistance exercise in normoxia and normobaric hypoxia. We hypothesised that hypoxia would augment the acute leukocyte and cytokine responses.

## MATERIALS AND METHODS

### Experimental Design

A single-blinded randomised trial was used to investigate the effects of resistance exercise in normobaric hypoxia on the acute leukocyte responses in older adults. The experimental methods used in this study are previously described, where we showed that performing hypoxic resistance exercise induces a safe but significant reduction in oxygen saturation to a minimum of 89.1 ± 1.2% (mean ± SD) [32]. Participants were closely monitored during their training session to ensure that their oxygen saturation did not drop below 80%. In brief, participants completed a single bout of supervised resistance exercise in either normobaric hypoxia or normoxia. Participants were randomly allocated to hypoxia or normoxia by a random number generator.

### Subjects

Twenty healthy, recreationally active males (n = 12) and females (n = 8) aged 60–70 years were recruited into the study (Normoxia; mean ± SD: age 64.0 ± 2.6 years, body mass 71.9 ± 13.8 kg, BMI 23.9 ± 2.6 kg · (m^2^)^−1^, Hypoxia; mean ± SD: age 65.9 ± 3.5 years, body mass 70.7 ± 14.3 kg, BMI 24.9 ± 3.6 kg · (m^2^)^−1^, n = 6 males and 4 females per group).

Statistical power for the study was calculated as part of a larger study [32]. Here, a power analysis was performed to demonstrate that the study was adequately powered to detect changes in our primary outcome measures (leukocyte). The analysis was performed using a priori in G*Power through repeated measures analysis of variance (ANOVA), within-between factors (the F test; Version 3.1.9.4; Universität Kiel, Germany) using our previous study that showed the immune response of young adults to a bout of resistance exercise in normobaric hypoxia [35]. Neutrophils were selected as the largest leukocyte population and 2 hours post-exercise was selected as the most important time point. Cohens d was calculated and transformed into effect size f. The parameters were; Alpha error probability (0.05), power (1-β error probability; 0.80), groups (two), number of measurements (two; pre-exercise and 2 hours post-exercise), standard correlation among repeated measures (0.5), non-sphericity correction (one). Therefore, to detect an effect size (f) of 0.721 using a two-way repeated measures ANOVA for mean neutrophil count, a minimum of eight participants were required per group.

Experimental procedures were approved by the Deakin University Human Research Ethics Committee (ID: 2016–308). The work was carried out in accordance with the Code of Ethics of the World Medical Association (Declaration of Helsinki). After the nature, purpose, risks and benefits of the study were explained, participants gave written informed consent to participate in the study.

### Procedures

Normobaric hypoxia was achieved in an environmental tent using a normobaric hypoxic generator (Pulford Systems, Australia), as previously described [32]. Briefly, the ambient air was enriched with nitrogen to reduce the FiO_2_ to 14.4%, the equivalent altitude of approximately 3,300 m above sea level [36]. To blind participants from their group allocation, individuals in the normoxic group also completed their session in the environmental tent with the generator set to FiO_2_ 20.93%. We have previously shown that with this level of hypoxia and exercise intensity, older adults were unable to consistently identify their environmental conditions [32]. Environmental conditions in the exercise physiology laboratory were maintained at 19°C with small fluctuations in air pressure.

### Training Session

After a 5-minute warm up on a stationary bicycle, participants performed four whole body exercises at 70% of their calculated 1RM, in the following order; leg extension, pectoral fly, standing row and squat. The method used to calculate 1RM was previously reported by our group [32]. Participants performed 4 sets of 10 repetitions for each exercise with a 1 minute rest between sets and a 2 minute rest between exercises. The session lasted approximately 50–60 minutes and participants exited the environmental tent after the last squat exercise, so that their recovery was completed in normoxia. Blood was sampled from an antecubital vein at the following time points; rest, 0, 15, 30, 60, 120, 180 minutes and 24 hours post-exercise. At each time point, 5 mL of venous blood was collected in an EDTA vacutainer (BD, USA). To ensure that the load performed by the normoxic and hypoxic groups was equivalent, the total work was calculated by summing the repetitions and weights lifted for each exercise and each participant. The total work was then averaged across the normoxic and hypoxic groups and analysed using an independent samples t-test.

### Haematology

A full blood count was performed on the EDTA-treated whole blood samples using a haematology analyser (DhX500, Beckman Coulter, Australia) to quantify the concentration of total leukocytes, neutrophils, lymphocytes, monocytes, eosinophils basophils and platelets. Samples were run in duplicate on the same day as blood collection. The haematology analyser was regularly cleaned and a 3-point calibration was performed before testing each sample batch. The % coefficient of variation (%CV) for leukocyte, lymphocyte, monocyte, neutrophil, eosinophil, basophil and platelet measurements for the data collection period was 1.2, 3.7, 4.0, 1.8, 15.1, 10.5 and 8.2% respectively. Given that plasma can shift between the blood and extracellular space with exercise, plasma volume shift was accounted for using the Dill and Costill method [37].

### Flow cytometry

Leukocyte subpopulations were characterised using flow cytometry with three colour analysis (FacsCanto II, BD Biosciences, Australia; FACSDIVA v9.0 software). All fluorescent dye-conjugated antibodies were sourced from BD Biosciences and underwent titration to determine the optimal volume of undiluted stock solution. Venous blood was analysed pre-exercise (rest) and 0, 180 minutes and 24 hours post-exercise using two panels. For each panel, 100 µL of EDTA treated whole blood was added to a 10 mL tube containing the relevant antibodies (Supplementary table 1). Samples were incubated at room temperature for 15 minutes in the dark. Two mL of 1 × lysing solution (buffered solution with < 15% formaldehyde and < 50% diethylene glycol; BD Biosciences, Australia) was added to each sample and incubated at room temperature for 10 minutes in the dark. Samples were centrifuged at 300 × g for five minutes and the supernatant was discarded. After washing, cells were resuspended in 2 mL of phosphate buffered saline (PBS), centrifuged at 300 × g for five minutes and the supernatant was discarded. Finally, cells were resuspended in 200 µL of PBS and stored at 4°C until analysis later that day using a FacsCanto II flow cytometer. Cytometer setup and tracking beads (CST; BD Biosciences, San Jose, CA, USA) were regularly run through the machine to confirm that the median fluorescent intensity of antigen positive high, intermediate, and low populations was comparable across the course of the study. The flow cytometer collected 10,000 lymphocytes from each sample, averaging approximately 60,000–90,000 events in total. Compensation was completed using single colour controls to minimise spectral overlap of the antibodies. FITC (CD45), APC-Cy7 (CD8), BV480 (CD14) and BV421 (CD45RA) were compensated using leukocytes and APC (CD3), PE (CD4) and PE-Cy7 (CD19) were compensated using commercial compensation beads (Anti-Mouse Ig, κ / Negative Control (FBS) Compensation Particles Set; BD Biosciences, Australia). All samples were run in duplicate, with a %CV of 3.44% for panel 1 and 3.31% for panel 2.

**SUPPLEMENTARY FIGURE 1:**
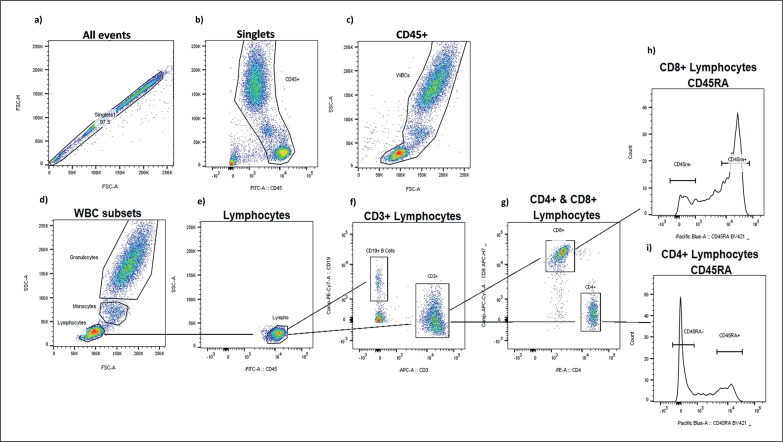
Panel one flow cytometry gating strategy, including all events (a), singlets (b), CD45^+^ (c), white blood cell (WBC) subsets (d), lymphocytes (e), CD3^+^ lymphocytes (f), CD4/CD8^+^ Lymphocytes and their expression of CD45RA (h, j). Populations analysed include total lymphocytes, lymphocyte subpopulations (B lymphocytes and T lymphocytes) and the two subpopulations of T lymphocytes; CD4^+^ T helper cells and CD8^+^ T cytotoxic cells. CD4^+^ and CD8^+^ T cells were stained with CD45RA as a crude marker of cell senescence.

The gating strategy firstly identified single cells (singlets) using forward light scattering (FSC) properties (Supplementary Figure 1a). Subsequently, the side scattering (SSC) of cells was plotted against CD45 to gate out any CD45 negative cells (CD45^−^; Supplementary Figure 1b). After using a single gate for leukocytes (Supplementary Figure 1c), the leukocyte subpopulations (granulocytes, monocytes and lymphocytes) were then visually identified using their FSC and SSC properties (Supplementary Figure 1d). For panel one, lymphocytes were isolated (Supplementary Figure 1e), then B and T lymphocytes were subsequently identified using CD3 and CD19 (Supplementary Figure 1f). Two subsets of T lymphocytes were identified using CD4 and CD8; T helper cells (CD4^+^) and T cytotoxic cells (CD8^+^; Supplementary Figure 1g). The expression of CD45RA on CD4^+^ and CD8^+^ T cells was used as a basic indicator of cell senescence, where naïve T cells are typically CD45RA^+^ and senescent T cells are CD45RA^−^ (Supplementary Figure 1h, i) [38].

**SUPPLEMENTARY FIGURE 2:**
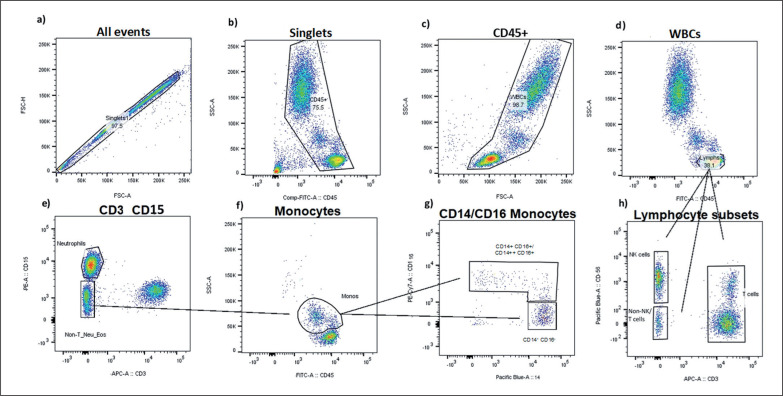
Panel two flow cytometry gating strategy, including all events (a), singlets (b), CD45^+^ (c), white blood cell (WBC) subsets (d), CD3/CD15 (e), monocytes (f), CD14/CD16 monocytes and lymphocyte subsets (h). Populations analysed include NK cells, total monocytes and the two monocyte subpopulations; CD14^+^ CD16^−^ monocytes (classical monocytes) and CD14^+/++^ CD16^+^ monocytes (grouped here as non-classical monocytes).

For panel two, monocyte populations were initially identified using CD3 and CD15 (Supplementary Figure 2e), then confirmed using CD45 (Supplementary Figure 2f). The subpopulations of monocytes were identified using CD14 and CD16 (Supplementary Figure 2g). Due to a low number of monocyte cells in each sample, it was not possible to definitively identify the three monocyte sub-populations; Classical (CD14^++^ CD16^−^), non-classical (CD14^+^ CD16^++^) and intermediate (CD14^++^ CD16^+^). For this reason, monocytes were identified either as classical (CD14^+^ CD16^−^) or non-classical (CD14^+^ CD16^+^), meaning that the small number of intermediate cells were likely included in the non-classical population. In panel two, NK cells were also identified using CD3 and CD56 antibodies (CD3^−^ CD56^+^).

### Cytokine analysis

Venous plasma samples were analysed in duplicate at the following time points; pre-exercise (rest), 0, 180 minutes and 24 hours post-exercise. A high sensitivity human cytokine Milliplex T cell panel (MPHSTCMAG28SK06; Merck Millipore, Abacus Dx, Australia) was used to quantify the protein expression of cytokines. Kits were designed for the simultaneous measurement of IL-1β, IL-4, IL-6, IL-8, IL-10 and TNFα. The assay was conducted in accordance with the manufacturer’s instructions, where standards were prepared for the high sensitivity range, equating to 0.49–2000 pg/mL for IL-1β, 1.83–7500 pg/mL for IL-4, 0.18–750 pg/mL for IL-6, 0.31–1250 pg/ mL for IL-8, 1.46–6000 pg/mL for IL-10 and 0.43–1750 pg/mL for TNFα. The average intra-assay %CV for IL-1β, IL-4, IL-6, IL-8, IL-10 and TNFα was 5.6, 6.8, 5.9, 4.6, 6.1, 6.1%, and the average interassay %CV was 3.2, 5.5, 2.0, 4.4, 1.1, 2.3% respectively. The minimum detection limit for IL-1β, IL-4, IL-6, IL-8, IL-10, and TNFα was 0.14, 1.12, 0.11, 0.13, 0.56 and 0.16 pg/mL respectively. If values were below the minimum detectable limit they were assigned the minimum detectable limit. There were a small number of participants excluded from the analysis due to having > 50% of measurements below the minimum detectable limit; IL1β (n = 2), IL-4 (n = 0), IL-6 (n = 1), IL-8 (n = 2), IL-10 (n = 1) and TNFα (n = 1).

### Statistical Analysis

Statistical analysis was completed using SPSS software (IBM SPSS 26, Chicago, IL) and graphed using GraphPad software (Prism 8.00, California USA). Data were assessed for normal distribution using a Shapiro Wilk test and an independent samples t-test was conducted to detect any between-group differences at pre-exercise (resting). Any variables that failed the Shapiro-Wilk test for normality underwent Log_10_ transformation prior to analysis. Analysis of the resting values revealed that leukocytes were different between groups at pre-exercise (rest). Therefore, the acute leukocyte responses to resistance exercise in hypoxia were analysed with an ANCOVA for the main effects of TIME (seven levels; 0, 15, 30, 60, 120 and 180 minutes, 24 hours post-exercise), GROUP (two levels; normoxia, hypoxia) using two covariates (sex, resting values). Sex was included as a covariate to account for known differences in leukocytes between males and females. For the leukocyte variables generated by flow cytometry (NK cells, B lymphocytes, T lymphocytes, CD4^+^ cells, CD8^+^ cells, CD45RA expression, CD14^+^ CD16^−^ monocytes and CD14^+^ CD16^+^ monocytes) there were four levels of TIME (pre-exercise and 0, 180 minutes and 24 hours post-exercise). If a TIME × GROUP interaction was present, a Bonferroni post-hoc test was used to evaluate the effect of hypoxia on individual time points. Data are presented as adjusted mean ± SD.

Given that the ANCOVA used the first resting value as a covariate (pre-exercise), it was not possible to determine the main effects of TIME across the normoxic and hypoxic groups. Therefore, a separate ‘time course analysis’ was performed using GROUP (two levels; normoxia, hypoxia), TIME (8 levels; pre-exercise, post 0, 15, 30, 60, 120 and 180 mins, 24 hrs) and COVARIATE (sex). The ANCOVA included pairwise comparisons on the pooled group data (estimated marginal means for TIME), where the resting (pre-exercise) value was compared to the post 0, 15, 30, 60, 120, 180 mins and 24 hrs values with Bonferroni confidence interval adjustment. This secondary analysis allowed comparison of the acute effects of exercise alone on the leukocyte responses, irrespective of group allocation.

Inflammatory cytokines showed no baseline (resting) or sex differences between hypoxia and normoxia, and were therefore analysed using an ANOVA for the main effects of TIME (four levels; pre-exercise and 0, 180 minutes and 24 hours post-exercise) and GROUP (two levels; normoxia, hypoxia). Significance was set at p < 0.05.

## RESULTS

**FIG. 1:**
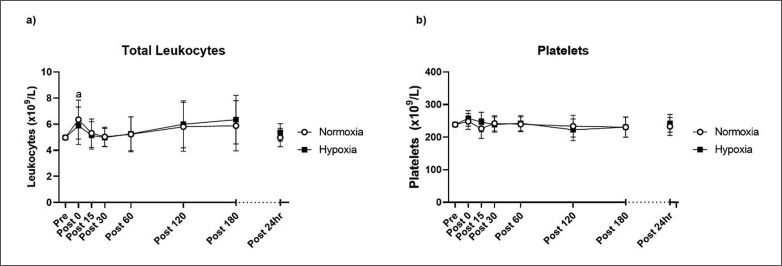
Total leukocytes (a) and platelets (b) pre- and post-exercise. Values are adjusted means ± SD (n = 10). ^a^ Significantly different from pre-exercise (p < 0.05).

The total work performed during the resistance exercise bout was not statistically different between normoxic and hypoxic groups (p = 0.388; independent samples t-test). After accounting for plasma volume shifts, sex and baseline differences, there was no TIME × GROUP interaction for total leukocytes (p = 0.853) or platelets in the 24 hours following the training session (p = 0.674; Figure 1a, b). The time course analysis showed that, irrespective of group allocation, total leukocytes increased immediately post-exercise (p = 0.032) compared to pre-exercise. Leukocytes then returned close to baseline and were not different to the pre-exercise values up to 24 hours post-exercise. Platelet counts did not change over the 24 hour measurement period (p > 0.05).

**FIG. 2:**
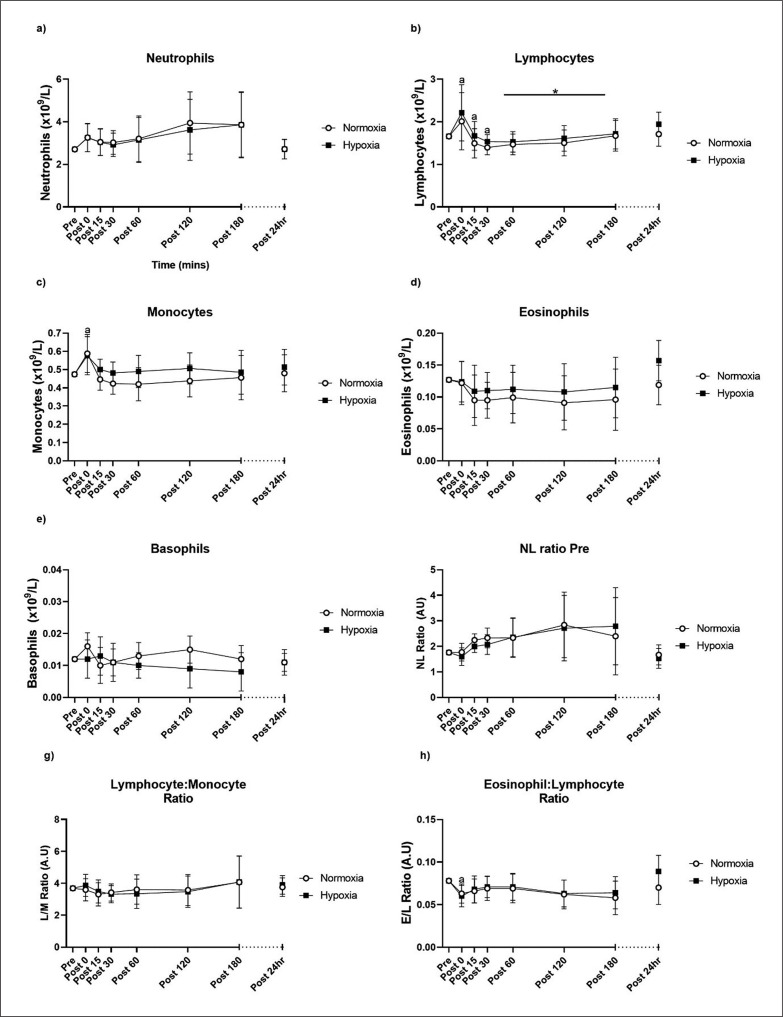
Neutrophils (a), lymphocytes (b), monocytes (c), eosinophils (d) and basophils (e), neutrophil-lymphocyte ratio (f), lymphocyte-monocyte ratio (g) and eosinophil-lymphocyte ratio pre- and post-exercise. Values are adjusted means ± SD (n = 10). * Main GROUP effect (p < 0.05). ^a^ Significantly different from pre-exercise (p < 0.05).

There were no TIME × GROUP interactions present for any of the five leukocyte subsets measured in the 24 hours following the training session; neutrophils (p = 0.994), lymphocytes (p = 0.995), monocytes (p = 0.421), eosinophils (p = 0.747) or basophils (p = 0.408), nor the ratio of neutrophils-lymphocytes (p = 0.906), lymphocytes-monocytes (p = 0.971) or eosinophils-lymphocytes (p = 0.235; Figure 2). However, there was a main GROUP effect for lymphocytes following the training session (p = 0.035), where lymphocyte concentrations were higher in hypoxia compared to normoxia. Monocytes, eosinophils and basophils showed no main GROUP effect. The time course analysis showed that, irrespective of group allocation, neutrophils showed a near-significant increase immediately post-exercise (p = 0.055), though were not different from pre-exercise at any other timepoint. There was an increase in lymphocytes immediately post-exercise (p < 0.001), followed by a decrease below pre-exercise levels at 15 (p < 0.001) and 30 minutes post-exercise (p = 0.018), returning close to pre-exercise values up to 24 hours post-exercise. Monocytes increased immediately post-exercise compared to pre-exercise (p = 0.009) and did not differ at any other timepoint. Eosinophils, basophils and the lymphocyte-monocyte ratio did not change over the 24 hours post-exercise. The neutrophil-lymphocyte ratio was increased above pre-exercise values at 15 and 30 minutes post-exercise (p < 0.001, p = 0.008, respectively). The eosinophil-lymphocyte ratio increased decreased post-exercise (p < 0.01) compared to pre-exercise, but was not different at any other timepoint.

**FIG. 3:**
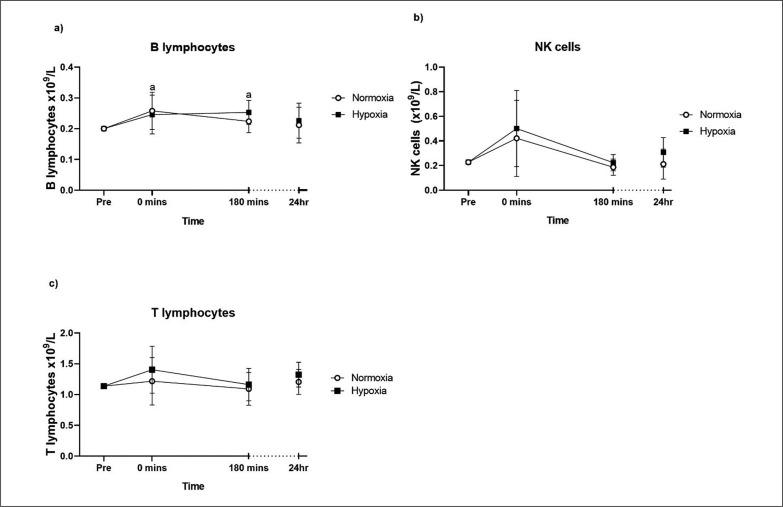
B lymphocytes (a), NK cells (b) and T lymphocytes (c) pre- and post-exercise. Values are adjusted means ± SD (n = 10). ^a^ Significantly different from pre-exercise (p < 0.05).

**FIG. 4:**
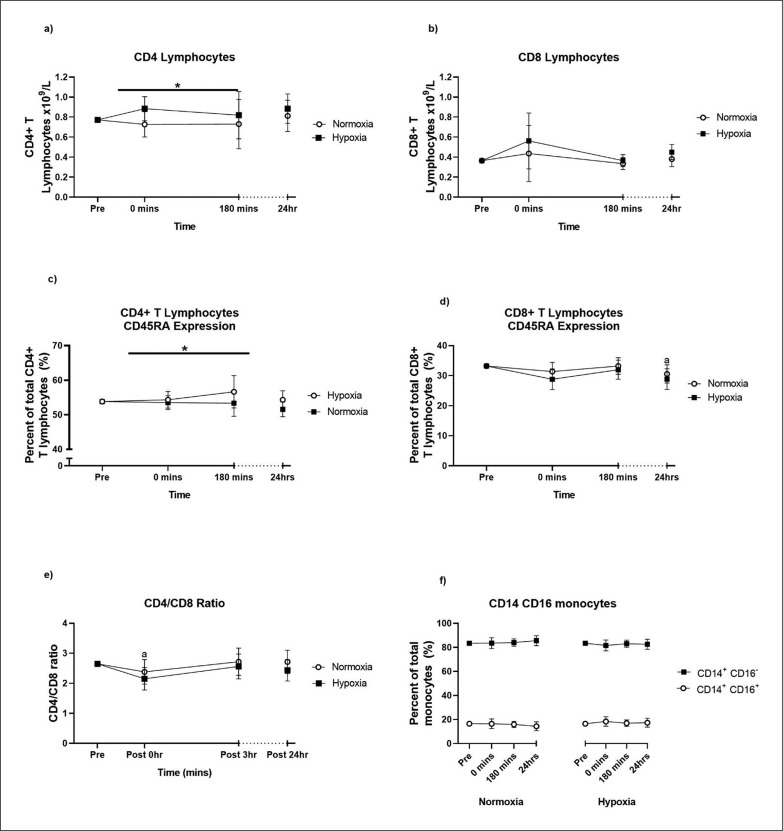
CD4^+^ T helper cells (a), CD8^+^ T cytotoxic cells, the subsets of CD45RA^+^ and CD45RA^−^ T cells (c, d), the CD4/CD8 ratio and the subsets of CD14^+^ CD16^−^ (classical) and CD14^+^ CD16^+^ (non-classical/intermediate) monocytes (f) pre- and post-exercise. Values are adjusted means ± SD (n = 10). * Main GROUP effect (p < 0.05). ^a^ Significantly different from pre-exercise (p < 0.05).

There were no TIME × GROUP interactions for the lymphocyte subpopulations; B lymphocytes (p = 0.549), NK cells (p = 0.795) and T lymphocytes (p = 0.885; Figure 3a, b, c), nor were there any main effects of GROUP. However, the analysis of T cell subpopulations showed a main GROUP effect, where the CD4^+^ T cell responses were higher in hypoxia compared to normoxia (p = 0.046; Figure 4a). CD4^+^ T helper cells also showed a main GROUP effect on CD45RA expression (p = 0.044), where there was a higher proportion of CD45RA^−^ CD4^+^ T cells in hypoxia compared to normoxia (Figure 4c). For the acute CD8^+^ T cytotoxic cell response and their expression of CD45RA, there was no main effect of GROUP or TIME following training session (Figure 4b, d). There was no main GROUP effect on the CD4:CD8 ratio in the 24 hours following the training session (Figure 4e). For the acute CD14^+^ CD16^−^ (classical) monocyte and the non-classical/intermediate (CD14^+^ CD16^+^) monocyte response there was no main GROUP effect following the training session (Figure 4f). The time course analysis showed that, irrespective of group allocation, B lymphocytes were increased at 0 minutes (p = 0.033) and 3 hours (p = 0.010) post-exercise compared to pre-exercise. NK cells were near-significantly increased at 0 minutes post-exercise compared to pre-exercise (p = 0.063). T lymphocytes (CD4^+^ and CD8^+^) and the expression of CD45RA on CD4^+^ T lymphocytes did not change in the 24 hours post-exercise. The proportion of CD45RA^+^ CD8^+^ T cells was lower 24 hours after the exercise bout (p = 0.041).

**FIG. 5:**
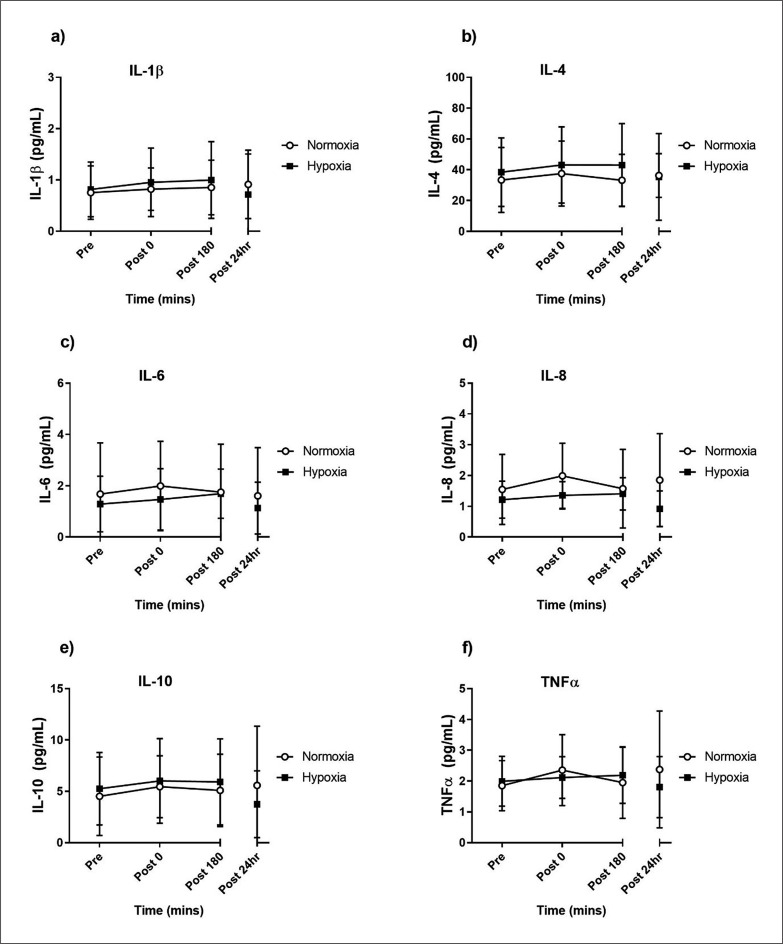
Acute Interleukin-1 beta (IL-1β; a) Interleukin-4 (IL-4; b), Interleukin-6 (IL-6; c), Interleukin-8 (IL8; d), Interleukin-10 (IL-10; e) and tumor necrosis factor alpha (TNF-α; f) pre- and post-exercise. Values are mean ± SD (n = 10).

The acute inflammatory cytokine responses to resistance exercise were measured at rest and 0, 180 minutes and 24 hours following the training session. The acute IL-1β, IL-4, IL-6, IL-8, IL-10 and TNFα responses did not show a TIME × GROUP interaction following the training session (p = 0.894, p = 0.236, p = 0.174, p = 0.072, p = 0.062, p = 0.259 respectively; Figure 5). The time course analysis showed that, irrespective of group allocation, there were no changes in cytokine concentrations in the 24 hours following the exercise bout (p < 0.05).

## DISCUSSION

Older adults experience blunted physiological adaptations to exercise [39] and reduced resistance to pathogens [40], both of which contribute to the declining health of ageing individuals. Systemic hypoxia augments the leukocyte response to resistance exercise in young adults [35], yet this response remained uncharacterised in older adults. Therefore, this study characterised the acute leukocyte and cytokine responses of older adults to a single bout of resistance exercise in hypoxia.

Analysis of circulating neutrophils, monocytes, eosinophils and basophils showed no effect of hypoxia in the 24 hours following the resistance training protocol. An acute increase in monocytes following exercise is typical, possibly due to increased sheer stress in the vasculature and monocyte release from bone marrow [41, 42]. We expected hypoxia to further increase the systemic monocyte response as acute hypoxia can reduce monocyte migration from the systemic circulation into peripheral tissues [43]. We also expected a larger neutrophil response to the hypoxic training protocol, given that young adults show a greater neutrophil response 120 and 180 minutes after resistance training in FiO_2_ 14.4%, compared to normoxic controls [35]. Older adults are generally thought to have blunted neutrophil responses to acute exercise compared to young adults [8], and it appears that this is not reversed by exposure to systemic hypoxia.

We demonstrated that older adults have higher lymphocyte numbers following resistance exercise in hypoxia compared to normoxia, that persists for 24 hours post-exercise. Considering that ageing typically reduces lymphocyte concentrations following exercise, the older adults exposed to hypoxia in this study may have partially restored their lymphocyte response. Hypoxia (FiO_2_ 14.4%) does not elicit a greater lymphocyte response in young adults when performing a similar resistance exercise protocol to the current study [35]. However, young adults do show the classic lymphocytosis immediately post-exercise followed by a modest decrease below baseline for approximately 3 hours post-exercise [35]. In the present study, the peak in lymphocytes immediately post-exercise in the older adults was approximately 2–2.5 × 10^9^/L, whereas the younger adults in our previous study peaked at approximately 3.5–4.0 × 10^9^/L. Although lymphocytes were not fully restored to that of young adults, it shows that hypoxia could benefit older adults and increase lymphocyte numbers following resistance exercise. It appears that the lymphocytopenia that typically occurs in the hours post-exercise was less pronounced in the hypoxic group compared to normoxia, suggesting if anything, that the older adults may have been less susceptible to pathogens in the systemic circulation. The exact mechanism causing the greater lymphocyte response in hypoxia is unknown. Although adrenaline [44], noradrenaline [45], growth hormone [46] and cortisol [47] are known recruiters of lymphocytes, we have previously shown that older adults do not show a greater concentration of these hormones in the 60 minutes following hypoxic resistance exercise, compared to normoxia [48]. Future studies would benefit from utilising both young and older participants to compare the lymphocyte responses to resistance exercise in hypoxia.

Analysis of the T lymphocyte subsets showed that exercising in hypoxia, compared to normoxia, elevated the proportion of circulating CD4^+^ T helper cells over the 24 hours post-exercise. It is difficult to elucidate the cause of this response, given that the number of T cells in circulation can be influenced by many factors including the initial rate of T cell influx from the periphery into the bloodstream and the subsequent rate of T cell movement back into peripheral tissues [49]. The number of T cells in the circulation can also be influenced by stress-induced apoptosis of T cells and the entry of naïve T cells from the thymus [50, 51].

We also showed a higher proportion of CD45RA^−^ CD4^+^ T cells in hypoxia in the 24 hours following the training session, when compared to normoxia. Although CD45RA is only a crude marker for cell senescence, it is possible that hypoxia differentially regulates the movement of CD4^+^ T cell phenotypes between peripheral tissues and the systemic circulation. It is possible that hypoxia caused greater recruitment of senescent CD4^+^ T cells into the bloodstream or greater apoptosis of naïve CD4^+^ T cells, given that a single bout of intense cycling in 12% O_2_ increased CD4^+^ T cell apoptosis through the caspase-9 pathway in young males [52]. These potential mechanisms require further investigation with a greater number of cell senescence markers. Despite the effects of hypoxia on the CD4^+^ T cell response, hypoxia did not affect the acute CD4:CD8 T cell ratio, CD8^+^ T cytotoxic response or the proportion of CD45RA^+^ CD8^+^ T cells. Resistance exercise alone elicited a decrease in the CD4:CD8 ratio immediately post-exercise that is consistent with moderate-intensity resistance exercise in older adults [53].

The inflammatory cytokines did not show any effects of hypoxia, nor did they change over the 24 hours post-exercise. This result is likely due to the variable nature of cytokine measurements [54] and a relatively small sample size in this study. Glycogen depletion is known to increase systemic IL-6 following exercise [55], therefore we had expected the increased reliance on carbohydrate metabolism with hypoxic resistance exercise to elevate IL-6 levels post-exercise. The exercise session may have been insufficient in both intensity and/or duration to induce a greater IL-6 response in hypoxia. Although the acute cytokine responses to resistance exercise in hypoxia were previously uncharacterised in older adults, systemic hypoxia exposure generally upregulates inflammatory pathways through transcription factors such as NF-кβ and hypoxia inducible factor 1 (HIF-1) [56, 57]. In young adults, resting exposure to normobaric hypoxia for 6.5 hours increases systemic levels of TNFα and IL-6 [58]. The only study to characterise the systemic inflammatory responses of young adults to acute resistance exercise in hypoxia found an increase in IL-6 four hours after exercise, but no changes to IL-4, IL-8, IL-10 or TNFα when compared to normoxia [59]. Future research with larger sample sizes is needed to determine the functional impact of these cytokine responses to resistance exercise in hypoxia, including the potential relationship between inflammatory cytokines and immune cell recruitment.

### Strengths and Limitations

The inclusion of frequent blood samples between 0 minutes and 3 hours post-exercise enabled a thorough characterisation of the leukocyte responses of older adults to resistance exercise in hypoxia. Although the inclusion of males and females was important for the generalisation of results to the older adult population, the sample size could be expanded to more definitively determine the leukocyte and cytokine responses to hypoxic resistance exercise in both older males and females. The inclusion of more leukocyte senescence markers would have also strengthened the study.

## CONCLUSIONS

Normobaric hypoxia amplifies the acute lymphocyte response to a single bout of resistance exercise in recreationally active, non-resistance trained older adults. Although these changes could confer enhanced immune function, more research is needed to clarify the causes and implications of these responses to resistance exercise in hypoxia. The potential implications of this response to lymphocyte function is of great interest in future research. Our findings show that hypoxia does not detrimentally affect the leukocyte response of older adults to resistance exercise and that it may enhance their lymphocyte responses to exercise. From a practical perspective, health professionals can prescribe hypoxic resistance exercise to older adults without any detriment to the acute leukocyte or cytokine response.

## Figures and Tables

**SUPPLEMENTARY TABLE 1:** Antibodies and flow cytometer settings for panel one and panel two. All antibody quantities listed were taken neat from the undiluted stock solution.

**Panel one**						
**Laser**	**Optical Filters**	**Markers**	**Fluorochrome**	**Amount (µL)**	**Cat No.**	**Clone**

Blue (488 nm)	530/30	CD45	FITC	10	555482	HI30
	585/42	CD4	PE	10	555335	RPA-T4
	780/60	CD19	PE-Cy7	2.5	557835	SJ25C1

Red (633 nm)	660/20	CD3	APC	10	555347	UCHT1
	780/60	CD8	APC-Cy7	2.5	560179	SK1

Violet (405 nm)	450/50	CD45RA	BV 421	2.5	562885	HI100

**Panel two**						
**Laser**	**Detector/PMT**	**Markers**	**Fluorochrome**	**Amount (µL)**	**Cat No.**	**Clone**

Blue (488 nm)	530/30	CD45	FITC	10	555482	HI30
	585/42	CD15	PE	10	555402	HI98
	780/60	CD16	PE-Cy7	2.5	557744	3G8

Red (633 nm)	660/20	CD3	APC	10	555347	UCHT1

Violet (405 nm)	525/40	CD14	BV480	2.5	566141	Mφp9
	450/50	CD56	BBV421	2.5	562752	NCAM16.2
